# Career-related teacher support as a secure base: an attachment theory model of career adaptability and future work self-salience among university students

**DOI:** 10.3389/fpsyg.2025.1679208

**Published:** 2025-09-12

**Authors:** Yuan Zhang, Ying Yu, Zhigang Wang

**Affiliations:** Business School, Zhuhai College of Science and Technology, Zhuhai, Guangdong, China

**Keywords:** attachment theory, CRTS, career adaptability, FWSS, PGO, university students

## Abstract

**Introduction:**

Global economic turmoil has intensified the youth employability crisis, with a 12.6% unemployment rate, leading to reduced future work self-salience (FWSS) among university students. Career education remains fragmented, often neglecting teachers’ dual role as attachment figures and providers of industry-relevant support. This study proposes that career-related teacher support (CRTS) enhances students’ career adaptability (CA) and FWSS based on attachment theory. Additionally, it examines how performance goal orientation (PGO) moderates these relationships.

**Methods:**

A nationally stratified random sample of 391 undergraduates from three university tiers in China completed questionnaires. Variables were assessed using validated Chinese scales. Mediation and moderation analyses were conducted using Hayes’ PROCESS macro in SPSS with 5,000 bootstrap resamples and 95% confidence intervals.

**Results:**

Six of seven hypotheses (H1–H6) were supported. CRTS enhances FWSS and CA, with CA mediating the relationship between CRTS and FWSS. PGO exhibited a paradoxical dual role: it weakened the effect of CRTS on CA while strengthening the effect of CA on FWSS.

**Discussion:**

This study demonstrates that CRTS functions as a secure base, enhancing CA and FWSS. CA accounted for 34.12% of the total effect of CRTS on FWSS. PGO’s paradoxical moderating effect suggests that while it weakens the link between CRTS and CA, it strengthens the path from CA to FWSS. These findings extend attachment theory into higher education, offering practical implications for integrating adaptability-focused modules into curricula and tailoring teacher support to counteract youth unemployment in uncertain labor markets.

## 1 Introduction

The global economic downturn—driven by the lingering effects of the coronavirus disease (COVID-19) pandemic, escalating geopolitical conflicts, and rapid technological disruption—has precipitated a youth employability crisis of unprecedented scale. According to the International Labour Organization (2024), global youth unemployment currently stands at 12.60%, which is more than twice the 5% general unemployment rate. Career development has transitioned from a linear, predictable pathway to a complex process characterized by ambiguity and uncertainty in this condition of instability (Canzittu, 2022; Hughes and Niu, 2021).

In this context, university students are increasingly experiencing what career theorists describe as “vocational paralysis”—a psychological gridlock in which economic constraints intersect with existential uncertainty (McInnes, 2016), leading to chronically diminished Future Work Self-Salience (FWSS). This condition is further intensified by the emergence of “slow employment,” a growing trend in which graduates intentionally delay entering the workforce due to a lack of clarity, confidence, or alignment between their personal goals and available opportunities (Wang and Li, 2022). Slow employment often signals deeper challenges related to career identity formation and decision-making capacity rather than representing a temporary postponement (Wang and Li, 2022).

Despite the urgency of these challenges, current university career support systems remain fragmented and inadequate (Wang and Li, 2022; Yuen et al., 2018). Career education is frequently disconnected from disciplinary curricula, often limited to generic workshops or one-time counseling sessions that lack integration with students’ academic experiences or emerging professional identities (Lim et al., 2024). Additionally, career services tend to concentrate on senior-year students, leaving underclassmen without early, formative guidance during critical periods of career identity development. This reactive, rather than proactive, approach contributes to students’ disorientation and lack of preparedness upon graduation (Wang et al., 2024b).

Compounding these systemic gaps is the underutilization of university teachers as pivotal agents in students’ career development. Although substantial research has explored the influence of teachers on academic achievement, their role in addressing career ambiguity has been largely overlooked - a paradox given their unique dual capacities: professional authority (rooted in industry expertise and real-world experience) and relational security (providing emotional support and mentorship). These capacities align fundamentally with attachment theory’s concept of a “secure bases,” which promotes exploration and resilience amid uncertainty (Bowlby, 1988).

Another theoretical ambiguity persists regarding the directional relationship between career adaptability (CA) and future work self-salience (FWSS). Some studies posited CA as an antecedent of FWSS (e.g., Guan et al., 2017; Taber and Blankemeyer, 2015), arguing that CA facilitates the development of FWSS to pursue desirable future careers. In contrast, other research suggested FWSS serves as a precursor to CA (e.g., Lu and Jia, 2022; Zeng et al., 2024), proposing that individuals with a clear future work self are more inclined to engage in proactive career behaviors and exhibit higher adaptability. Our proposed sequential mediation pathway (CRTS → CA → FWSS) aims to reconcile this contradiction by empirically testing and establishing the predominant direction of influence within this specific supportive context.

To bridge these gaps, this study employs attachment theory as its foundational lens. Unlike existing frameworks that largely focus on individual self-regulation or career narratives, the attachment theory highlights the relational dynamics between students and teachers as pivotal to career development. It portrays teachers as “secure-base” figures who simultaneously fulfill the roles of professional mentors and emotional caregivers, thereby cultivating both CA and FWSS. This approach yields several innovations: (1) it redirects emphasis from intrapsychic processes to interpersonal mechanisms; (2) it integrates emotional containment with skill scaffolding to explain how teacher support fosters adaptive career outcomes; and (3) it introduces a novel mediation pathway (CRTS → CA → FWSS) to reconcile existing theoretical contradictions.

Through this secure-base relationship, students develop CA - the psychological toolkit for navigating labor market volatility (Savickas and Porfeli, 2012). Enhanced CA subsequently crystallizes FWSS, enabling clearer, more confident career planning. This framework extends attachment theory in higher education by prioritizing relational security as a catalyst for career exploration and offers a more holistic understanding of career development processes.

Crucially, university students’ performance goal orientation (PGO) is introduced as a cognitive moderator that is believed to influence the extent to which career support is translated into career clarity. Although PGO has traditionally been associated with maladaptive outcomes in academic settings, its role may differ meaningfully within the context of career development and the competitive labor market, where demonstrating competence and securing positive evaluations are essential. This study addresses longstanding theoretical debates regarding the paradoxical effects of PGO, which can hinder and facilitate learning and motivation depending on contextual factors (Elliot, 1999; Vandewalle et al., 2001). Examining the moderating role of PGO in the relationship between CRTS, CA, and FWSS provides a more nuanced understanding of how career development processes shape individual differences.

## 2 Literature review and hypotheses development

### 2.1 Career-related teacher support and future work self-salience of university students

Originating with Bowlby (1969) and empirically elaborated by Ainsworth (1978), attachment theory asserts that early relationships with caregivers shape internal working models—cognitive-emotional frameworks that guide individuals’ expectations of themselves and others, enabling adaptive functioning through the development of affect regulation and the provision of a secure base (Bowlby, 1988; Mikulincer and Shaver, 2016).

CRTS refers to the multifaceted assistance of educators in students’ career development, encompassing emotional encouragement, skill-building, and labor-market guidance, such as industry-specific training, career-planning discussions, and mentorship that enhance self-efficacy and decision-making (Wong et al., 2021; Zhang et al., 2021).

FWSS is defined as the extent to which individuals can clearly and vividly envision their hoped-for future selves in work roles, reflecting meaningful career aspirations (Strauss et al., 2012). This vivid self-representation functions as an internal bridge between one’s current self-concept and future professional behavior, motivating proactive career actions such as skill development, job searching, and career planning (Strauss et al., 2012). Empirical studies have shown that the FWSS positively predicts employment quality and job performance among college students and new employees (Guan et al., 2013). As a motivational resource, FWSS enables individuals to set clear career goals, seek feedback, and adjust their behaviors in response to career challenges, making it a critical factor in career development (Lu and Jia, 2022).

In educational contexts, teachers function as secondary attachment figures, providing both emotional security and professional guidance (Pianta, 1999; Verschueren and Koomen, 2012). Empirical studies have shown that supportive teacher-student relationships promote students’ exploratory behavior, executive functioning, and academic resilience, while also buffering against anxiety and uncertainty (Bergin and Bergin, 2009; Hamre and Pianta, 2001; Lin et al., 2024).

When this attachment-based framework is extended to career development, university teachers are conceptualized as dual-role attachment figures whose influence is exerted through two complementary pathways (Mayseless, 1996).

First, in the professional identity pathway, teachers actively scaffold the formation of FWSS by providing industry-specific modeling and experiential learning opportunities. Through competency mapping and strategic internships, they offer concrete, real-world reference points that allow students to visualize and concretize their possible future selves (Mayseless, 1996; Zhang et al., 2018). This professional scaffolding helps students convert abstract career interests into tangible, achievable professional identities, thereby enriching the detail and credibility of their FWSS.

Second, in the emotional containment pathway, teachers strengthen their students’ FWSS by creating a secure psychological base that encourages identity exploration and reduces defensive avoidance. By transforming anxiety into validated exploration, reflecting unrecognized strengths, and normalizing trial-and-error learning, they mitigate the emotional barriers that inhibit self-projections into the future (Mayseless, 1996; Song et al., 2022). This support enables students to engage in future-oriented identity work with greater confidence and psychological safety, allowing them to develop a more stable, salient, and elaborated future work self.

Self-determination theory (SDT) offers complementary insights into how teacher support facilitates student development (Ryan and Deci, 2000). SDT posits that such support nurtures students’ basic psychological needs for autonomy, competence, and relatedness (Banerjee and Halder, 2021). Crucially, satisfying these needs enhances intrinsic motivation and strengthens students’ capacity to envision future possibilities. While SDT primarily focuses on motivational processes, its emphasis on supportive interpersonal relationships as essential for growth closely aligns with attachment theory. Thus, SDT provides a reinforcing framework that explains how CRTS contributes to the development of a clearer, more salient, and motivating FWSS by cultivating the necessary psychological conditions for proactive, future-oriented identity construction. Additionally, the study by Greco and Kraimer (2020) demonstrated that career mentoring contributes to the construction of a professional identity, a process which is closely aligned with the development of FWSS.

By integrating these perspectives, it is proposed that teacher career support is conceptualized as a secure base that strengthens university students’ FWSS, leading to the first hypothesis:

*H1*: CRTS positively influences the FWSS scores of university students.

### 2.2 University students’ career-related teacher support and career adaptability

CA refers to an individual’s capacity to manage and respond effectively to career transitions and challenges, encompassing four key dimensions: confidence, control, curiosity, and concern (Savickas and Porfeli, 2012). It reflects a person’s readiness to make informed career decisions, adapt to evolving environments, and construct a meaningful professional trajectory. Empirical research has demonstrated that CA is positively associated with academic performance, job search success, and overall career satisfaction (Ghosh and Fouad, 2017; Guan et al., 2013). For university students, it serves as a vital psychological resource for navigating modern labor market uncertainties and demands.

Attachment theory positions teachers as secure-base providers whose dual roles foster CA through distinct yet complementary pathways (Bowlby, 1969, 1988). Teachers promote self-reliance in their professional identity role by offering industry-specific skill scaffolding, such as commercial negotiation simulations that enhance self-efficacy in problem-solving (Wong et al., 2021). Simultaneously, caregivers nurture social competence through relational modeling of professional behaviors (e.g., cross-disciplinary collaboration frameworks) and emotional containment of career-related anxieties. This dual-role synergy cultivates courage to explore by providing psychologically safe, low-risk opportunities for adaptive experimentation.

Empirical research supports this framework, showing that teachers’ social support is significantly associated with positive developmental outcomes across multiple domains. Previous studies have demonstrated that teacher support enhances students’ academic performance, motivation, school engagement, and career exploration behaviors (Creed et al., 2006, 2009; Lent et al., 2019; Rogers et al., 2008; Rosenfeld et al., 2000). Furthermore, teacher support has been linked to the development of cooperative behaviors that are essential for workplace success (Chiu and Chow, 2011; Ryan and Shim, 2012; Sun and Shek, 2012).

These findings align with the premise of attachment theory that secure-base relationships facilitate the exploration and development of competence. When teachers simultaneously supply domain mastery tools and relational security, a secure base is transformed into adaptive resources, allowing career support to be directly converted into enhanced adaptability for navigating volatile occupational landscapes. Therefore, it is posited that.

*H2*: CRTS positively influences the CA of university students.

### 2.3 Career adaptability and future work self-salience of university students

The relationship between CA and FWSS has been a central focus in the career development literature; however, theoretical perspectives on their directionality remain divided. Drawing on career construction theory (CCT) and self-regulatory theory, some scholars argue that FWSS acts as a motivational driver, encouraging individuals to cultivate psychological resources, such as CA, to bridge the gap between their current and ideal future selves (Savickas and Porfeli, 2012). Conversely, other studies indicate that FWSS emerges as a consequence of CA, with highly adaptable individuals demonstrating more proactive job-search behaviors and possessing clearer future work identities (Guan et al., 2014; Strauss et al., 2021). This ongoing theoretical tension highlights the need for alternative frameworks to clarify the mechanisms that link these two constructs. To bridge this gap, this study employs attachment theory as its foundational lens.

The CA was conceptualized as the adaptive internal working model rooted in attachment-based security, functioning as a key psychosocial resource that facilitates four core processes of reality-calibration. First, career confidence promotes proactive experimentation—such as conducting informational interviews—which sharpens self-awareness by testing vocational hypotheses (Savickas, 1997). Second, career control supports intentional self-regulation, allowing students to align current skill development with future self-concepts (Savickas, 1997). Third, career curiosity encourages exposure to diverse occupational narratives, helping to correct self-concept distortions through social mirroring (Savickas, 1997). Finally, career coping enables adaptive iteration, guiding students in systematically refining their FWSS schemas following setbacks, thus removing unrealistic elements and enhancing goal realism (Savickas, 1997).

Collectively, through continuous environmental feedback loops, these capacities transform abstract career aspirations into grounded professional identities. By converting challenges into opportunities for strategic refinement, CA sustains dynamic future self-narratives. These iterative adjustments to personal strengths and perceived opportunities help align vocational goals with real-world conditions. This psychological scaffolding directly enhances FWSS by facilitating the crystallization of evidence-based identity. Thus, it is posited that.

*H3*: The CA of university students positively influences their FWSS scores.

### 2.4 Mediating role of career adaptability

Attachment theory conceptualizes CA as the transformative engine through which teacher-provided career support enhances students’ clarity about their future work. Teachers create a secure base through mastery of the dual-role scaffolding-transmitting industry through their professional identity and offering exploratory safety through their caregiver role. Students internalize this dual support as an adaptive internal working model, operationalized as the CA. This adaptability converts external resources into personal career insight through three integrated processes: (1) knowledge internalization, which translates industry insights into self-reliance competencies; (2) safety operationalization, which enables risk-calibrated exploration through emotional containment; and (3) identity crystallization, which refines professional self-concepts through social feedback. CA bridges CRTS and FWSS by continuously mediating between relational security and self-definition, transforming abstract vocational possibilities into concrete, evidence-based professional representations. This position adopts adaptability as the core mechanism through which attachment-based security is translated into agentic career identity formation. Thus, it is posited that.

*H4*: CA mediates the relationship between CRTS and FWSS among university students.

### 2.5 The moderating effect of performance goal orientation

Performance goal orientation (PGO) is defined as a pattern of achievement motivation in which an individual’s primary aim is to demonstrate competence and ability relative to others, to gain favorable judgments, and to avoid negative evaluations of their capability (Dweck, 1986; Elliot, 1999). It is characterized by a preoccupation with normative success, social comparison, and “proving oneself” in the eyes of others (Dweck, 1986).

Traditionally, PGO has been associated with maladaptive outcomes such as surface learning and performance-avoidant behaviors in academic contexts (Alhadabi and Karpinski, 2020). However, in the domain of career development and preparation for the competitive job market, PGO may play a significantly different and potentially adaptive role.

The modern labor market, characterized by intense competition for prestigious positions and the necessity to proactively present oneself to potential employers, creates an environment where performance-oriented mindset may be strategically advantageous for enhancing CA and FWSS.

Previous research reveals a complex relationship between PGO and career outcomes. While learning goal orientation is consistently associated with positive career development, findings regarding PGO have been mixed (Porath and Bateman, 2006). For instance, Creed and Hennessy (2016) found that PGO is associated with higher career exploration, stronger career aspirations, greater use of career-related strategies, and the setting of high goals. However, individuals high in PGO may also employ less effective strategies, be less inclined to seek feedback, and operate under the belief that their abilities are fixed (Creed and Hennessy, 2016). This would in turn reduce their intrinsic motivation and reward (Creed and Hennessy, 2016).

This apparent paradox underscores the context-dependent nature of PGO outcomes. Its emphasis on demonstrating competence and gaining external validation aligns closely with the performance-driven demands of the job market, suggesting that PGO may function differently in career versus academic contexts. To reconcile these contradictory effects, this study examines the moderating role of PGO in the relationship between CRTS, CA and FWSS. This investigation aims to offer a more nuanced understanding of how PGO operates within career development processes.

The achievement goal theory posits that performance-oriented students prioritize demonstrating competence over engaging in exploratory learning (Dweck, 1986; Gerhardt and Brown, 2006). When PGO is high, students tend to filter teacher support instrumentally, seeking validation for pre-existing goals while dismissing developmental resources that may threaten their self-concept, such as cross-disciplinary internships that carry a risk of perceived failure (Vandewalle, 2003). This selective engagement inhibits the translation of support into CA. Conversely, students with low PGO are more receptive to scaffolding from teachers, as an opportunity for skill development and adaptive growth (Porath and Bateman, 2006). Thus, PGO functions as a cognitive gatekeeper: high orientation induces defensive filtering of support inputs, weakening adaptability outcomes, whereas low orientation fosters holistic resource integration, amplifying the impact of teacher support on career adaptation. Accordingly, it is hypothesized that.

*H5*: University students’ PGO negatively moderates the relationship between CRTS and CA.

The PGO amplifies the relationship between the CA and FWSS through its goal-congruent processing function. Students with elevated PGO strategically convert adaptability resources into targeted self-definition inputs: they transform exploratory experiences into validated evidence of competence, selectively filter social feedback for goal-relevant signals, and align skill development with demonstrable strengths (Creed and Hennessy, 2016; Lent et al., 2016). This cognitive prioritization accelerates professional identity crystallization by anchoring adaptive behaviors to performance standards (Creed and Hennessy, 2016). Conversely, students with low PGO apply adaptability resources more diffusely, lacking selective filtration that sharpens self-concept clarity, thereby weakening their ability to generate clear self-projections for future work (Lent et al., 2016). Thus, PGO functions as a cognitive accelerator, intensifying the adaptability—FWSS link by distilling complex career explorations into coherent, evidence-based self-narratives in which every adaptive act becomes intentional data for vocational identity formation (Creed and Hennessy, 2016; Super, 1990). Accordingly, it is posited that.

*H6*: University students’ PGO positively moderates the relationship between CA and FWSS.

The mediating effect of CA between teacher career support and FWSS is either amplified or attenuated by PGO through distinct cognitive pathways. High-PGO students act as targeted converters: their focus on demonstrating competence enables them to strategically transform teacher-provided support into adaptability gains while also filtering adaptive experiences into goal-congruent future self-projections. This dual-stage optimization—leveraging skill acquisition support and distilling adaptability into strength-based narratives—intensifies the mediating effect (Creed and Hennessy, 2016; Lent et al., 2016). Conversely, low-PGO students display more diffuse cognitive processing, which limits their ability to translate support into adaptability and to consolidate those experiences into coherent vocational identities (Lent et al., 2016). Thus, PGO functions as a cognitive accelerator that strengthens the indirect CRTS → CA → FWSS pathway when it is elevated but weakens it when it is diminished by regulating the effective conversion of attachment-based security into agentic career clarity (Creed and Hennessy, 2016; Super, 1990). Therefore, it is posited that.

*H7*: PGO moderates the mediating role of the CA of university students in the relationship between CRTS and FWSS, such that the mediating effect is stronger and weaker when PGO is high and low, respectively.

**Figure 1:**
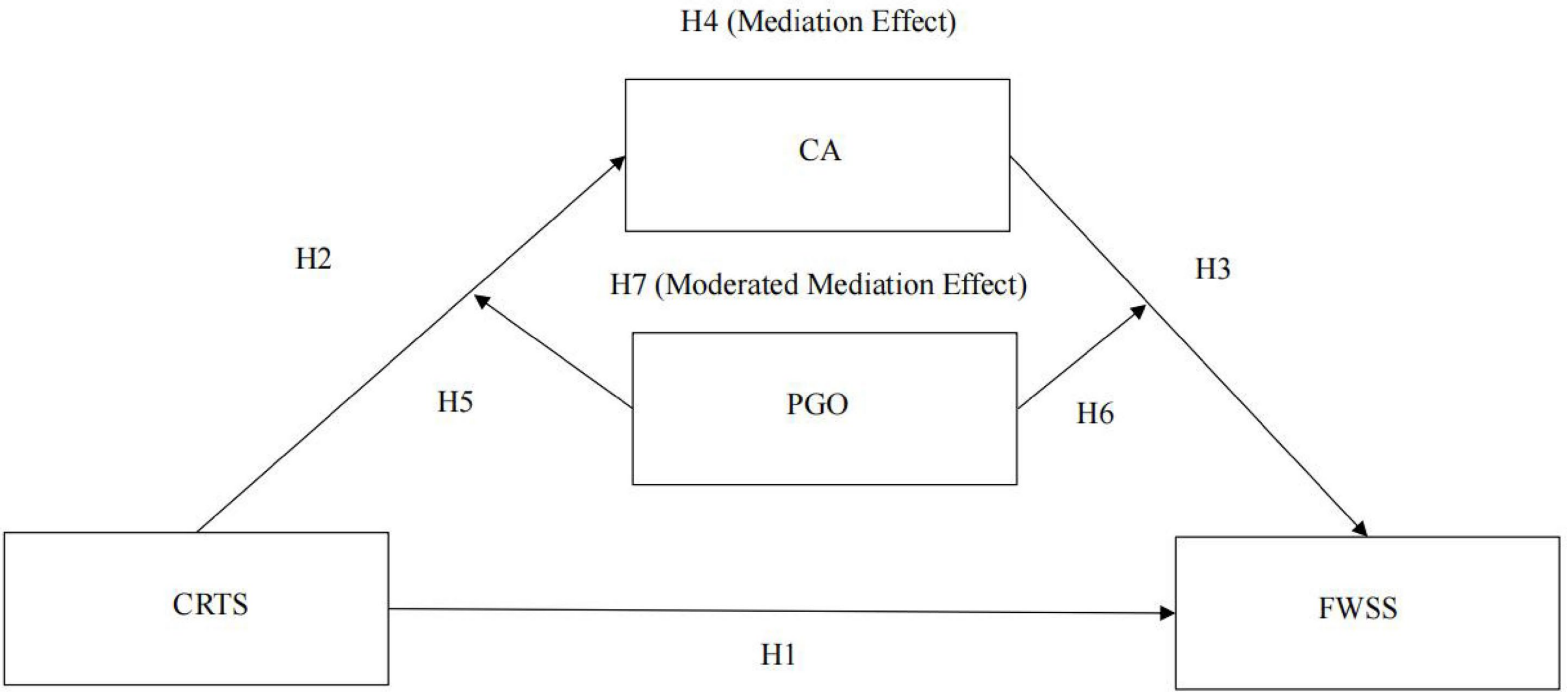
Theoretical model. CRTS = career-related teacher support; CA = career adaptability; PGO = performance goal orientation; FWSS = future work self-salience.

Figure 1 illustrates the hypothesized model

## 3 Methods

### 3.1 Sample

A stratified random sampling approach was employed through a three-phase process to ensure national representativeness across diverse socioeconomic and educational contexts. First, regional stratification grouped China’s provinces into four socioeconomic clusters: (1) Coastal Developed Zones (e.g., Guangdong, Shanghai, Zhejiang); (2) Central Rising Economies (e.g., Hubei, Hunan, Shanxi); (3) Western and Northeastern Developing Regions (e.g., Sichuan, Chongqing, and Heilongjiang); and (4) Political-Educational Centers (Beijing and Tianjin). Second, institutional sampling involved randomly selecting two or three universities per region, weighted by enrollment size, to represent three institutional tiers: double first-class universities, regular undergraduate institutions, and vocational universities. Third, participants were recruited through alumni networks and student affairs offices using electronic links or QR codes. A quota sampling strategy was initially employed, targeting a gender distribution (40% male, 60% female) that reflects the composition of the national higher education student population.

Data collection for this study was carried out in March and April of 2025. Following data collection and subsequent screening, a total of 391 valid questionnaires were retained for analysis. The sample comprised students majoring in humanities and social sciences (*n* = 231, 59.08%), science and engineering (*n* = 119, 30.43%), and other fields such as interdisciplinary studies (*n* = 41, 10.49%). Participants were distributed across academic years as follows: freshmen (*n* = 94), sophomores (*n* = 145), juniors (*n* = 85), and seniors or above (*n* = 67). The final gender distribution consisted of 129 male students (32.99%) and 262 female students (67.01%). This deviation from the initial quota is common in online survey research and likely reflects the higher response rate typically observed among female participants, as well as the specific demographic profiles of the participating schools and majors. Institutional representation included double first-class universities (*n* = 41, 10.49%), local or private universities (*n* = 288, 73.66%), and vocational undergraduate institutions (*n* = 62, 15.85%). The specific distribution across each university is detailed in Table 1.

**Table 1:** Sample distribution across different university tiers and regions.

Region	University code	Institutional tier	N per university
Coastal developed zones	U01	T1	13
	U02	T2	90
	U03	T3	26
		Subtotal	129
Central rising economies	U04	T1	10
	U05	T2	95
	U06	T3	19
		Subtotal	124
Western and northeastern developing regions	U07	T1	8
	U08	T2	68
	U09	T3	17
		Subtotal	93
Political-educational centers	U10	T1	10
	U11	T2	35
		Subtotal	45

*N* = 391. T1 = double first-class universities, T2 = regular undergraduate institutions, T3 = vocational universities.

To address potential concerns regarding the stratified sampling design and its ability to capture cross-tier differences, a one-way analysis of variance (ANOVA) was conducted to examine variations in the core outcome variables across the three institutional tiers (T1: Double First-Class universities; T2: Regular undergraduate institutions; T3: Vocational universities).

Using SPSS 25.0, the results revealed no statistically significant differences in CA, (*F*_(2,388)_ = 1.529, *p* = 0.218), or FWSS (*F*_(2, 388)_ = 1.119, *p* = 0.328) across university tiers. *Post hoc* comparisons using the Turkey HSD test confirmed that none of the pairwise differences were significant (all *p* > 0.05). Specifically, for CA, the mean differences between T1 and T2 (*M*diff = −0.184), T1 and T3 (*M*diff = −0.124), and T2 and T3 (*M*diff = 0.060) were not statistically significant. Similarly, for FWSS, the mean differences between T1 and T2 (*M*diff = 0.187), T1 and T3 (*M*diff = 0.087), and T2 and T3 (*M*diff = −0.102) were all negligible and non-significant. This consistent pattern of non-significant results suggests that the mean levels of these psychological constructs are statistically equivalent across the different university tiers in our sample.

It should be noted that while the stratified sampling strategy aimed to capture national socioeconomic and institutional diversity, the final sample proportions, such as the higher representation of female participants and the distribution of academic majors, do not exactly match the demographic profile of the national university student population. Nevertheless, the primary objective of this study was not to provide precise descriptive estimates of population parameters, but to examine the relationship between psychological constructs. The theoretical model investigates core psychological relationships—between CRTS, CA and FWSS—which are hypothesized to be stable and meaningful across different demographic groups. Thus, the primary research aim was to test the validity of these proposed relationships rather than to quantify subgroup differences. Therefore, the identified mechanisms can be considered robust, and the findings regarding these core relationships remain both valid and interpretable.

### 3.2 Measurement

All the measurement scales were originally in English; thus, Brislin (1970)‘s translation and back-translation procedure were employed, and a 5-point Likert scale was used, ranging from 1 (“strongly disagree”) to 5 (“strongly agree”).

CRTS was assessed using a 6-item scale developed by Zhang et al. (2021), which demonstrated excellent internal consistency (*α* = 0.949). Sample items included “My teachers at my university help me develop my career values” and “My teachers at my university guide me to explore the outside world of work (e.g., professional categories).”

CA was measured using the 12-item Career Adapt-Abilities Scale–Short Form developed by Maggiori et al. (2017), which demonstrated excellent overall reliability (α = 0.940) and comprises four subscales—Career Confidence (*α* = 0.823), Career Control (*α* = 0.811), Career Curiosity (*α* = 0.862), and Career Coping (*α* = 0.858), with representative items such as “Thinking about what my future will be like” and “Observing different ways of doing things.”

FWSS was assessed using a 3-item scale developed by Strauss et al. (2012), which demonstrated high reliability (*α* = 0.904), with sample items including “I can easily imagine my future work self” and “The mental picture of this future is very clear.”

PGO was measured using a 6-item scale developed by Button et al. (1996), which demonstrated good reliability (*α* = 0.885), with sample items including “I prefer to do things that I can do well rather than things that I do poorly” and “The things I enjoy the most are the things I do the best.”

Students’ age, gender, and academic grade were statistically controlled. Gender was coded as a dummy variable with “1” representing male and “2” representing female. University type was also controlled, serving as a key proxy for a range of socioeconomic and institutional factors. In China, admission into elite universities (e.g., “Double First-Class” institutions) is highly competitive and often correlated with stronger prior academic preparation, which itself can be influenced by family socioeconomic status. Additionally, university type is intrinsically linked to institutional funding and resources, which are often tied to the economic development of the host province.

This measure was prioritized over direct individual socioeconomic status (SES; e.g., parental income or education) due to significant concerns about participant sensitivity and response bias, either of which could compromise data validity. The utilization of regional economic indicators (e.g., provincial GDP) was also carefully considered. However, defining the most relevant regional indicator presents a conceptual challenge, as students are influenced by both their household registration region (*hukou*, a system of household registration that ties access to public services to one’s registered location) and their current university region, which frequently differ substantially. University type was therefore employed as a pragmatic proxy that captures aspects of both students’ socioeconomic background and their current institutional and regional environment. University type was coded as “1″ for double first-class universities, “2″ for other universities, and “3″ for vocational universities.

### 3.3 Analytical approach

In line with Yu et al. (2021), Hayes’ PROCESS macro (Version 4.1, Models 4 and 58) was employed for hypothesis testing because of its superior ability to evaluate the theoretically specified dual-stage moderated mediation model. This method was preferred over structural equation modeling (SEM) because it effectively accommodates the hypothesized asymmetric moderation effects—specifically, the negative moderating influence of PGO on the CRTS → CA path (Stage 1) and its positive moderating effect on the CA → FWSS pathway (Stage 2). Model 58’s computational framework directly estimates these distinct moderation effects through interaction terms and calculates the integrative Index of Moderated Mediation within a single analytical process, thereby avoiding the parameter confounding often associated with latent interaction modeling in SEM.

## 4 Data analysis and results

### 4.1 Assessment of the common method variance

The Harman single-factor test was conducted to evaluate the impact of common method variance (CMV) on the study outcomes. This test involved an exploratory factor analysis in SPSS, including all items from both independent and dependent variables without factor rotation, to assess whether a single factor accounted for most of the variance. The first factor explained 46.955% of the total variance. According to the Harman test criterion, if a single factor explains more than 50% of the variance, CMV is considered a serious concern (Harman, 1960). Because the explained variance in this case was below the threshold, the CMV is unlikely to significantly bias the research findings.

Although the survey adopted a single-source, single-time design, several procedural remedies were implemented to mitigate the potential effects of CMV. First, respondents were not required to provide student IDs or any identifying information during data collection, thereby reducing social desirability bias. Second, the questionnaire included clear and detailed instructions to help participants understand each item accurately, thereby minimizing confusion and response bias. Third, irrelevant or filler items were interspersed between measurement items to obscure the purpose of the study and reduce hypothesis guessing, thereby minimizing demand characteristics and other potential biases.

In summary, although some CMV was detected, its impact on the research results was minimal, as the design-related control measures effectively mitigated potential bias, thereby supporting the findings’ reliability and validity.

### 4.2 Assessment of reliability and validity

Using SPSS 25.0, the Kaiser-Meyer-Olkin measure of sampling adequacy was calculated as 0.96, indicating strong intercorrelations among variables and the suitability of the data for factor analysis (Kaiser, 1974). Bartlett’s test of sphericity was significant (*χ^2^* = 9149.42, *df* = 351, *p* < 0.001), confirming that the correlation matrix significantly differed from an identity matrix and further supporting the appropriateness of factor analysis.

The composite reliability (CR) and average variance extracted (AVE) were computed for all constructs to assess internal consistency and convergent validity, following Fornell and Larcker’s (1981) criteria (CR > 0.70, AVE > 0.50), and the results indicated strong psychometric properties across the measurement model.

For CRTS, all six items demonstrated strong factor loadings (*λ* = 0.807–0.923), resulting in excellent convergent validity (AVE = 0.761) and CR = 0.950. Similarly, the three-item FWSS scale showed robust loadings (λ = 0.827–0.912), high reliability (CR = 0.905), and equally strong convergent validity (AVE = 0.761). The four CA sub-dimensions revealed acceptable to strong psychometric properties: career concern (λ = 0.712–0.824) showed good reliability (CR = 0.824) and valid convergence (AVE = 0.611); career control (λ = 0.708–0.811) yielded acceptable reliability (CR = 0.811) with slightly borderline convergent validity (AVE = 0.589); and career curiosity (λ = 0.793–0.865) and career confidence (λ = 0.776–0.845) both demonstrated excellent reliability (CR = 0.864 and 0.861) and convergent validity (AVE = 0.680 and 0.674, respectively).

The higher-order CA construct, which integrates the four sub-dimensions, demonstrated exceptional reliability (CR = 0.965) and convergent validity (AVE = 0.872), supported by strong second-order loadings (λ = 0.894–0.969), whereas the six-item PGO scale showed moderate-to-strong loadings (λ = 0.626–0.820), good reliability (CR = 0.887), and acceptable convergent validity (AVE = 0.569), although the relatively low loading of the first item (λ = 0.626), slightly reduced the overall AVE.

All constructs met the minimum psychometric standards, demonstrating sufficient reliability and convergent validity, as both career control (AVE = 0.589) and PGO (AVE = 0.569) exceeded the 0.50 threshold and showed acceptable CR (> 0.80). Future refinement could target the fourth item of career control (λ = 0.708) and the first item of PGO (λ = 0.626) to enhance scale performance.

Confirmatory factor analysis was conducted to evaluate the factorial structure of the measurement. The baseline model, comprising four distinct factors, demonstrated a good overall fit to the data (Table 2): *χ^2^* (314) = 845.398, *χ^2^/df* = 2.692, CFI = 0.935, TLI = 0.928, RMSEA = 0.066, and SRMR = 0.051. Based on the conventional criteria proposed by Hu and Bentler (1999), which recommend *χ^2^/df* < 3; CFI and TLI > 0.90, RMSEA < 0.08, and SRMR < 0.08—the model indices met all thresholds, indicating an acceptable and well-fitting measurement structure.

**Table 2:** Confirmatory factor analysis.

Models	Factors	*χ^2^*	*df*	*χ^2^*/*df*	CFI	TLI	RMSEA	SRMR
Baseline Model	4 Factors: CRTS, CA, PGO, FWSS	845.398	314	2.692	0.935	0.928	0.066	0.051
Model 1	3 Factors: CRTS, CA, PGO + FWSS	1606.761	317	5.07	0.843	0.827	0.102	0.095
Model 2	3 Factors: CRTS+CA, PGO, FWSS	2281.881	318	7.18	0.762	0.737	0.126	0.084
Model 3	2 Factors: CRTS+CA, PGO + FWSS	2750.523	320	8.60	0.705	0.676	0.139	0.090
Model 4	2 Factors: CRTS, CA + PGO + FWSS	2105.890	323	6.52	0.783	0.765	0.119	0.087
Model 5	1 Factor: CRTS+CA + PGO + FWSS	3347.839	324	10.33	0.633	0.602	0.154	0.105

*N* = 391. CRTS = career-related teacher support; CA = career adaptability; PGO = performance goal orientation; FWSS = future work self-salience. χ^2^ = chi-square, df = degree of freedom, CFI = comparative fit index, TIL = Tucker-Lewis Index, RMSEA = root mean square error of approximation, SRMR = standardized root mean square.

Alternative models with combined factors exhibited significantly poorer fit (Table 2), and this degradation in fit supports the four-factor structure’s discriminant validity, which was therefore retained for subsequent analyses.

### 4.3 Descriptive statistics and correlation analysis

Table 3 presents the means, standard deviations (SD), and bivariate correlations among all study variables. Several meaningful patterns emerged. First, the demographic variables showed expected interrelationships: age was significantly negatively correlated with gender (*r* = −0.106, *p* < 0.05), indicating that female students (coded as “2”) tended to be younger. Age also showed a strong positive correlation with grade level (*r* = 0.569, *p* < 0.01), consistent with typical academic progression, and a small but significant negative correlation with university type (*r* = −0.123, *p* < 0.05). All core research variables demonstrated significant positive intercorrelations. CRTS was strongly positively associated with CA (*r* = 0.609, *p* < 0.01), PGO (*r* = 0.474, *p* < 0.01), and FWSS (*r* = 0.541, *p* < 0.01). CA was also strongly correlated with both PGO (*r* = 0.630, *p* < 0.01) and FWSS (*r* = 0.517, *p* < 0.01). PGO and FWSS were moderately correlated (*r* = 0.313, *p* < 0.01). Gender was not significantly associated with any of the psychological variables, and university type was not correlated with any of the core constructs. Although grade level was strongly associated with age, it was unrelated to all key psychological variables. These correlation patterns offer preliminary support for the proposed conceptual model and underscore the relevance of including demographic variables as covariates in subsequent analyses to control for their potential confounding effects.

**Table 3:** Descriptive statistics and correlations.

Variables	Mean	SD	1	2	3	4	5	6	7	8
gen	1.670	0.471	–							
age	21.107	1.760	−0.106^*^	–						
unitype	2.054	0.511	0.074	−0.123^*^	–					
grade	2.320	1.022	0.055	0.569^**^	−0.062	–				
CRTS	3.457	0.828	−0.033	−0.004	0.045	−0.089	0.949			
CA	3.812	0.651	−0.018	0.028	0.034	0.065	0.609^**^	0.940		
PGO	3.748	0.671	−0.006	0.015	−0.034	0.033	0.474^**^	0.630^**^	0.885	
FWSS	3.149	0.840	−0.076	0.095	−0.013	0.017	0.541^**^	0.517^**^	0.313^**^	0.904

*N* = 391. ^*^p < 0.05, ^**^p < 0.01 (2-tailed). SD = standard deviation; gen = gender (1 = male, 2 = female); unitype = university type (1 = double-first class universities, 2 = other universities, and 3 = vocational universities); CRTS = career-related teacher support; CA = career adaptability; PGO = performance goal orientation; FWSS = future work self-salience. The numbers on the diagonal represent Cronbach’s α.

### 4.4 Hypothesis testing

#### 4.4.1 Testing of the mediation model

The mediation analysis using PROCESS Model 4 tested whether CA mediates the relationship between CRTS and FWSS, while controlling for gender, age, university type, and grade level.

Regarding H1, which posits a direct effect of teacher career support on university students’ FWSS, a significant total effect was observed: *β* = 0.543, *t* = 12.691, *p* < 0.001 (Table 4). Higher levels of career support for teachers are associated with greater clarity in students’ future work selves, thereby providing empirical support for H1.

**Table 4:** Regression results for the direct effects of CRTS on CA and FWSS.

Variable	Model 1: CA		Model 2: FWSS (Direct effect)		Model 3: FWSS (Full model)	
	*β*	*t*-value	*β*	*t*-value	*β*	*t*-value
Predictors						
gen	−0.013	−0.307	−0.050	−1.149	−0.046	−1.106
age	−0.056	−1.129	0.076	1.444	0.093	1.829
unitype	0.010	0.247	−0.022	−0.037	−0.026	−0.620
grade	0.153	3.103^**^	0.023	0.443	−0.022	−0.438
CRTS	0.621	15.454^***^	0.543	12.691^***^	0.358	6.833^***^
CA					0.298	5.722^***^
Model summary						
R	0.553		0.622		0.600	
R^2^	0.305		0.387		0.360	
*F*-value	33.848		48.563		35.990	

*N* = 391. ^**^p < 0.01, ^***^p < 0.001, (2-tailed). gen = gender (1 = male, 2 = female); unitype = university type (1 = double-first class universities, 2 = other universities, and 3 = vocational universities); CRTS = career-related teacher support; CA = career adaptability; FWSS = future work self-salience.

According to Table 4, CRTS had a strong positive effect on university students’ CA (*β* = 0.621, t = 15.454, *p* < 0.001), supporting H2. H3 was also supported, as CA significantly predicted FWSS (*β* = 0.298, t = 5.722, *p* < 0.001). This indicates that students with higher levels of CA reported clearer future work selves, even after controlling for teacher career support and relevant covariates.

When CA was controlled, the direct effect of CRTS on the FWSS of university students remained significant but was reduced in magnitude (*β* = 0.358, *t* = 6.833, *p* < 0.001). This finding indicates that while teacher career support continues to exert a direct influence on students’ clarity about their future work selves, part of its total effect is transmitted indirectly through the mediating role of CA.

The total effect of CRTS on the FWSS of university students was significant (Tables 5, *b* = 0.551, BootSE = 0.043, 95% CI [0.466, 0.637]). The indirect effect through CA was also statistically significant (Table 4, *b* = 0.188, BootSE = 0.033, 95% Boot CI [0.125, 0.255]). The completely standardized indirect effect was 0.185 (95% Boot CI [0.121, 0.252]), indicating that CA mediated a notable portion (34.12%) of the total effect of CRTS on FWSS. These results support H4 and highlight CA as a critical mechanism through which teacher career support shapes future-oriented career cognition among university students.

**Table 5:** Analysis of the mediating effect of CA.

Path	Effect	BootSE	95% LLCI	95% ULCI
Total effect of CRTS on FWSS	0.551	0.043	0.466	0.637
Direct effect of CRTS on FWSS	0.363	0.053	0.259	0.468
Indirect effect of CA	0.188	0.033	0.125	0.255

*N* = 391. Bootstrap *n* = 5,000. CRTS = career-related teacher support; CA = career adaptability; FWSS = future work self-salience.

LLCI = lower limit confidence interval, ULCI = upper limit confidence interval.

#### 4.4.2 Testing of the moderated mediation model

PROCESS Model 58 was employed to examine whether the PGO of university students moderates the effects of CRTS on CA and the effects of CA on FWSS.

A significant interaction was found between CRTS and PGO on CA, *b* = −0.082, *t* = −2.274, *p* < 0.05, indicating a moderating effect (Table 6). The positive association between CRTS and CA was stronger for students with low PGO than for those with high PGO. This pattern supports H5, indicating that higher PGO levels reduce the positive impact of CRTS on the CA of university students.

**Table 6:** Results of regression and conditional effect analyses for the moderation effect of PGO.

Variable	Model 1: CA (Mediator)		Model 2: FWSS (Outcome)	
	*b*	*t*	*b*	*t*
gen	−0.015	−0.311	−0.074	−1.010
age	−0.014	−0.870	0.048	2.016*
unitype	0.048	1.067	−0.055	−0.818
grade	0.067	2.450^*^	−0.016	−0.397
CRTS	0.328	10.415^***^	0.361	6.778^***^
CA			0.478	6.200^***^
PGO	0.420	10.896^***^	−0.097	−1.469
CRTS×PGO	−0.082	−2.274^*^		
CA × PGO			0.190	3.035^*^
Model summary				
R	0.733		0.615	
R^2^	0.537		0.379	
F-value	63.240^***^		29.081^***^	
Conditional indirect and direct effects at values of the moderator (PGO)				
	*b*	BootSE	LLCI	ULCI
Conditional effect analysis of CRTS → CA at PGO = Mean ± SD				
Mean − SD (3.077)	0.383	0.041	0.302	0.464
Mean (3.748)	0.328	0.032	0.266	0.390
Mean + SD (4.419)	0.273	0.038	0.198	0.349
Conditional effect analysis of CA → FWSS at PGO = Mean ± SD				
Mean − SD (3.077)	0.350	0.082	0.190	0.511
Mean (3.748)	0.478	0.077	0.326	0.629
Mean + SD (4.419)	0.605	0.094	0.421	0.789

*N* = 391. ^*^p < 0.05, ^***^p < 0.001, (2-tailed). SD = standard deviation. Gen = gender (1 = male, 2 = female); unitype = university type (1 = double-first class universities, 2 = other universities, and 3 = vocational universities); CRTS = career-related teacher support; CA = career adaptability; PGO = performance goal orientation; FWSS = future work self-salience. Bootstrap *n* = 5,000. LLCI = lower limit confidence interval, ULCI = upper limit confidence interval.

The interaction between CA and PGO significantly predicted FWSS (Table 6, *b* = 0.190, *t* = 3.035, *p* < 0.05). Further analysis revealed that the positive relationship between CA and FWSS strengthened as PGO increased. Specifically, CA significantly predicted FWSS at low PGO levels, whereas this effect was significantly stronger at high PGO levels. These findings support H6, indicating that PGO enhances the positive influence of CA on students’ self-clarity in future work.

**Figure 2:**
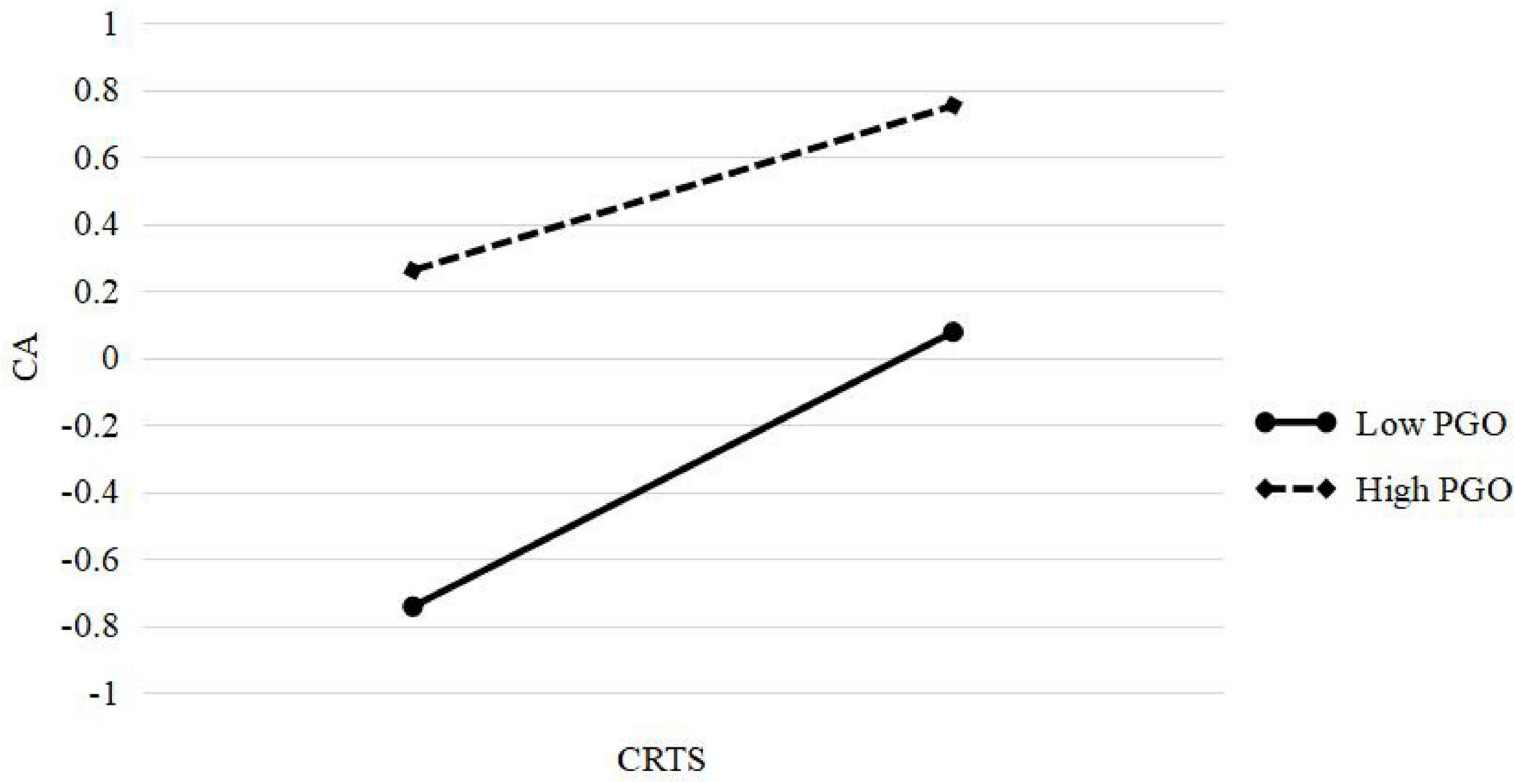
The moderation effect of university students’ PGO on the relationship between CRTS and CA. PGO = performance goal orientation; CRTS = career-related teacher support; CA = career adaptability.

**Figure 3:**
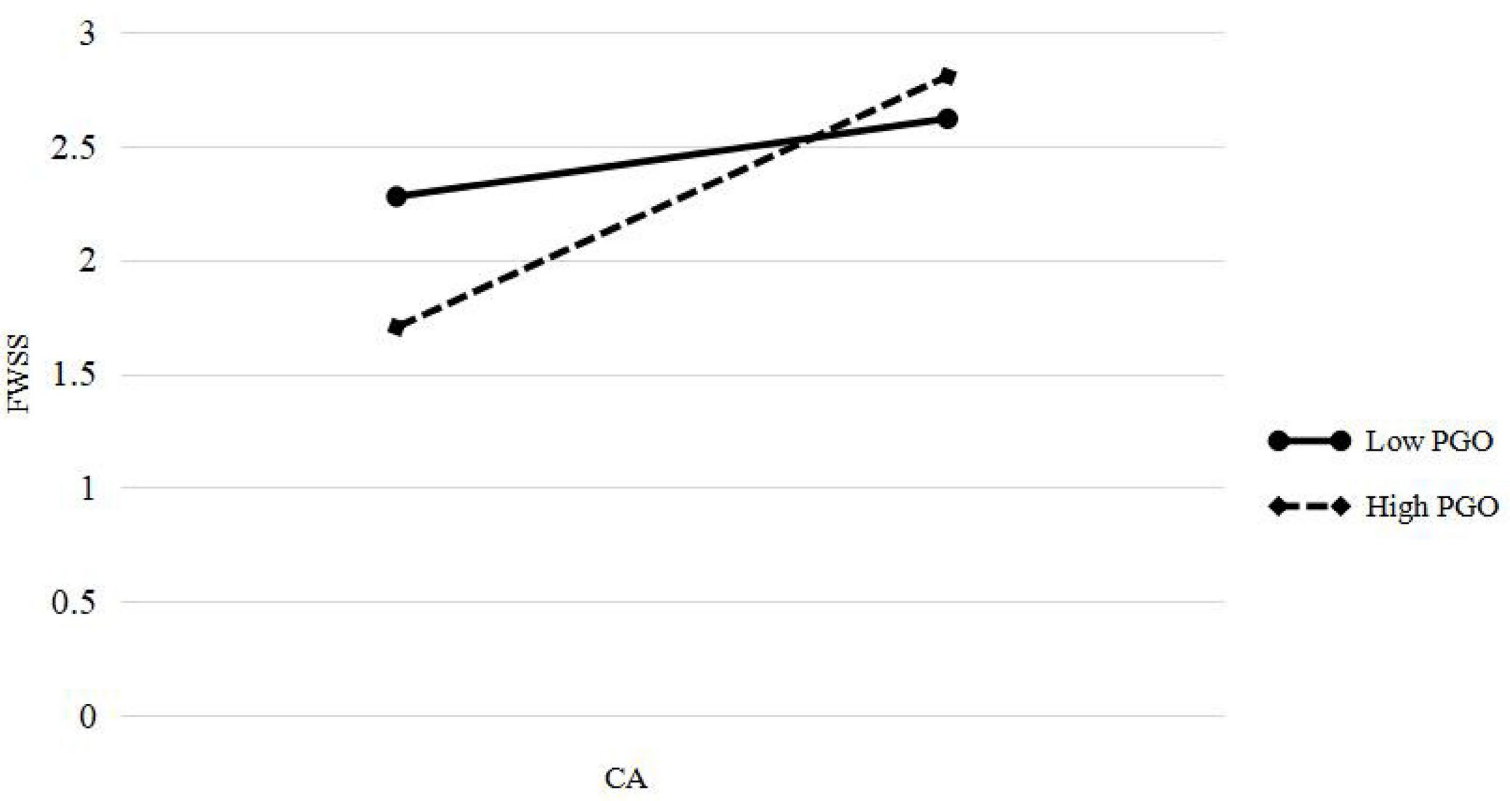
The moderation effect of university students’ PGO on the relationship between CA and FWSS. PGO = performance goal orientation; CA = career adaptability; FWSS = future work self-salience.

Moreover, simple slope analyses were conducted to illustrate the moderating role of PGO in two key pathways. First, when PGO was low (Mean – 1SD = 3.077), CRTS exerted a stronger positive effect on students’ CA (Figure 2). Second, when PGO was high (Mean + 1SD = 4.419), CA had a stronger positive effect on FWSS (Figure 3).

The results revealed that the mediating effect of CA on the relationship between teacher career support and FWSS was numerically stronger at higher levels of PGO, but this variation was not statistically significant (Table 7), thereby failing to support Hypothesis 7. Specifically, when PGO was low (Mean – 1SD = 3.077), the indirect effect was 0.134 (95% CI [0.070, 0.199]); at the mean level (3.748), the indirect effect increased to 0.157 (95% CI [0.104, 0.214]); and when PGO was high (Mean + 1SD = 4.419), the indirect effect was 0.165 (95% CI [0.104, 0.241]). Although the indirect effects (CRTS → CA → FWSS) were significant across all levels of PGO, pairwise contrasts revealed no statistically significant differences. None of the contrasts — between high and low, mean and low, or high and mean PGO levels — reached significance. These results indicate that the strength of the mediation effect did not significantly vary as a function of PGO.

**Table 7:** Results of the moderated mediation analysis: the indirect effect of CRTS on FWSS via CA at different levels of PGO.

PGO level	Indirect effect	BootSE	95% BootCI
Low (Mean − 1SD)	0.134	0.033	[0.070, 0.199]
Mean	0.157	0.028	[0.104, 0.214]
High (Mean + 1SD)	0.165	0.035	[0.104, 0.241]

Pairwise contrast	Difference	BootSE	95% BootCI
High vs. Low	0.031	0.040	[−0.037, 0.119]
Mean vs. Low	0.023	0.020	[−0.013, 0.064]
High vs. Mean	0.090	0.021	[−0.027, 0.056]

*N* = 391. SD = standard deviation. CRTS = career-related teacher support; CA = career adaptability; PGO = performance goal orientation; FWSS = future work self-salience. Bootstrap n = 5000.

In summary, six of the seven proposed hypotheses were empirically supported. CRTS directly enhanced FWSS (H1) and CA (H2), with CA serving as a significant mediator (H4). Additionally, PGO moderated key pathways: it weakened the effect of CRTS on adaptability (H5) but amplified the influence of adaptability on FWSS (H6). However, the moderated mediation effect proposed in H7 was not statistically supported.

## 5 Discussion

The findings of this study illuminate the psychological mechanisms through which CRTS enhances the FWSS of students, with CA serving as a key mediating pathway, and the moderation effect of PGO offers important theoretical and practical implications.

First, the significant direct effect of CRTS on FWSS confirms that career support functions as a foundational resource for fostering clarity about future careers. Students who perceive greater support from their teachers are more likely to form a clearer vision of their future work selves. This finding is consistent with previous research highlighting the importance of contextual support in shaping career-related cognition and behavior (Lent et al., 1994; Savickas, 2013).

In support of H2, CRTS significantly enhanced the CA of university students. This indicates that supportive teacher behaviors, such as providing guidance, encouragement, and access to resources, can strengthen students’ confidence, sense of control, curiosity, and coping strategies in career planning. This finding contributes to the literature on CA by highlighting the influential role of educational mentors in shaping students’ adaptive career behaviors (Song et al., 2022).

As predicted by H3, CA was a significant positive predictor of FWSS. Students with higher adaptability levels reported greater clarity regarding their future work selves, even after controlling for career support and demographic variables. This finding supports the view that CA is not only a valuable career resource but also a cognitive mechanism that enables individuals to form coherent and stable career identities (Gao and Li, 2025; Savickas and Porfeli, 2012).

Most importantly, the results confirmed H4 by demonstrating a significant indirect effect of CA on FWSS. This indirect pathway accounted for approximately 34.12% of the total effect, indicating that CA is a key mediating mechanism. These findings also underscore the pivotal role of CA within the career construction framework (Savickas, 2013). Students with greater adaptability resources appear better equipped to translate teacher support into concrete and coherent future self-images—a process that is especially crucial in uncertain career landscapes (Guan et al., 2017).

This study employed PROCESS Model 58 to examine the moderated mediating effects of PGO on the relationship between CRTS, CA, and FWSS, offering nuanced insights into how individual motivational orientations interact with contextual support to shape career development outcomes.

A significant interaction emerged between CRTS and PGO in predicting CA (*b* = −0.082, *p* < 0.05), supporting Hypothesis 5. Simple slope analysis revealed that the positive effect of career support on CA weakened as the PGO increased. Specifically, CRTS had a strong positive effect on CA at low PGO levels (*b* = 0.383, *p* < 0.001), whereas this effect was attenuated at high PGO levels (*b* = 0.273, *p* < 0.001). This indicates that students with high PGOs may be less responsive to teacher support in CA development. A plausible explanation is that individuals high in PGO, being more extrinsically driven, may view teacher support as less directly relevant to their goal attainment, thereby reducing its influence on their adaptive career behaviors.

The analysis identified a theoretically important pattern: PGO exerted a stronger direct influence on CA than CRTS. While teacher support significantly predicted CA (*b* = 0.328, *p* < 0.001), PGO demonstrated even greater predictive strength (*b* = 0.420, *p* < 0.001). This indicates that students’ intrinsic achievement motivation may play a more critical role than external support in shaping their adaptability. This finding aligns with the concept of self-regulatory primacy, where PGO functions as a cognitive filter that influences how students interpret and respond to career-related challenges, thereby affecting how effectively they use available resources (Elliot, 1999). Moreover, high-PGO learners are more likely to proactively transform environmental inputs, such as teacher support, into adaptability resources, whereas low-PGO students may passively receive such inputs without fully leveraging them. These finding challenges conventional resource-centric models by emphasizing that students’ engagement with support may be more consequential than the mere availability of support, in conjunction with the observed negative moderating effect of PGO on the relationship between CRTS and CA. This underscores the importance of accounting for individual goal orientation differences when designing career interventions, as the effectiveness of teacher support appears to vary according to students’ goal orientations.

The interaction between CA and PGO in predicting FWSS was statistically significant (*b* = 0.190, *p* < 0.05), supporting Hypothesis 6. Simple slope analysis indicated that the positive effect of CA on FWSS intensified as the PGO level increased. Specifically, CA had a moderate effect on FWSS at low PGO levels (*b* = 0.350, *p* < 0.001), whereas the effect was substantially stronger at high PGO levels (*b* = 0.605, *p* < 0.001). This pattern indicates that students with high PGOs are better able to translate their adaptive capacities into clearer future work selves. One plausible explanation is that high-PGO individuals are more focused on achieving specific goals (Elliot, 1999) and are thus more motivated to apply their adaptability resources toward building a coherent and goal-aligned career identity.

Consistent with a previous study, the indirect effect of CRTS on FWSS through CA was significant at all PGO levels, supporting the mediating role of CA, as posited in Hypothesis 4. However, the strength of this mediation did not vary significantly across PGO levels, resulting in the rejection of Hypothesis 7. Although the indirect effect was numerically stronger at high PGO levels (*b* = 0.165) than at low PGO levels (*b* = 0.134), pairwise comparisons of these indirect effects were not statistically significant. This finding indicates that while PGO may shape how CRTS influences career development processes, it does not significantly moderate the indirect pathway from CRTS to FWSS through CA.

One plausible theoretical explanation for this null finding may lie in the dual and potentially opposing roles that PGO plays at different stages. On one hand, students with high PGO may be more vigilant and responsive to CRTS at the initial stage, as they are motivated to gain mentors’ approval and demonstrate competence, thus potentially enhancing the effect of CRTS on CA. On the other hand, in the subsequent stage where CA translates into FWSS, high PGO may inhibit profound identity exploration due to fear of making mistakes or being negatively evaluated, thereby weakening the effect of CA on FWSS. These countervailing mechanisms – where PGO strengthens the first stages (CRTS → CA) but weakens the second (CA → FWSS) – may cancel each other out, resulting in a non-significant moderated mediation effect.

Moreover, CFA of the PGO scale produced a mixed pattern of fit indices: *χ^2^*/df = 7.092, RMSEA = 0.125, CFI = 0.954, TLI = 0.923, SRMR = 0.033. The elevated *χ^2^* and RMSEA values indicate some degree of model misfit, which is a not uncommon in scales containing items that are sensitive or subject to varied interpretations. By contrast, the CFI and TLI values exceeded the 0.90 threshold, and the SRMR was below 0.05, suggesting that the hypothesized factor structure is generally supported. The average variance extracted (AVE) was 0.569, slightly below the conventional 0.60 threshold, though composite reliability was acceptable (CR = 0.841). One item exhibited a relatively low factor loading (*λ* = 0.626). This combination of mixed fit indices and borderline convergent validity suggests the presence of measurement error in the PGO construct. Consequently, the non-significant moderating effect found for PGO in H7 should be interpreted with caution, as this measurement imperfection likely attenuated the estimated effects and reduced statistical power.

This finding suggests that PGO does not uniformly facilitate or hinder the mediating pathway. Its influence may be more complex and stage-specific. The distinctive effects of PGO at different phases of career development requires further research to better capture its nuanced regulatory role.

### 5.1 Theoretical contributions

This study advances the intersection of educational psychology and career development theory by offering four key contributions.

#### 5.1.1 Establishment of an attachment-based educational framework for career development

The reconceptualization of career support as a secure base within academic settings has been pioneered, thereby extending attachment theory into the career guidance of university students. Educators are positioned as catalysts for exploratory career behaviors, addressing significant gaps in relational, school-based vocational development approaches. Although previous studies on educational attachment have primarily focused on childhood and adolescence, it is demonstrated in this study that teacher-student relationships directly influence career outcomes during emerging adulthood. By framing teachers as secure-base providers who deliver both professional scaffolding and emotional support, attachment theory’s applicability is expanded beyond early developmental stages to encompass the ongoing formation of higher education career identification.

#### 5.1.2 Validation of the impact of career adaptability on FWSS through an attachment-based perspective

This study clarifies how teacher support facilitates vocational readiness by identifying CA as a key psychological mediator. An attachment-informed validation of the impact of adaptability on FWSS is offered, showing that CA functions as a relationally activated process and not merely as an individual competency. Consistent with attachment theory, security-based teacher support encourages exploratory behavior, which mobilizes adaptability resources to help students construct agentic, future-oriented career identities. This study integrates social cognitive career theory with educational psychology to demonstrate how supportive classroom relationships transform adaptability into a dynamic, psychosocial mechanism that guides career transitions and fosters future self-construction.

#### 5.1.3 Positioning relational foundations in the formation of professional identity

FWSS is repositioned as an outcome of security-guided career exploration, challenging static trait models and demonstrating how teacher-facilitated psychological safety is used to dynamically shape professional identity formation, thereby reframing FWSS as a teachable cognitive-affective capacity rooted in educational relationships.

#### 5.1.4 Validation of cultural and contextual career education

Data were collected from 11 universities in 4 regions in China to ensure socio-economic diversity. This approach ensures a comprehensive representation of the varied cultural and employment contexts in China. The findings advance culturally responsive career theory applications in non-Western context and challenge universalist assumptions in educational psychology. By emphasizing the distinct cultural and employment contexts in China, the study provides a more accurate understanding of career development processes compared to Western settings.

### 5.2 Practical implications

#### 5.2.1 Integrating career adaptability training into disciplinary curricula

Currently, university career education is often detached from students’ major-specific coursework, creating a gap between academic instruction and transferable skills development. To address this issue, CA training should be integrated into disciplinary curricula. Modules on artificial intelligence ethics and career path case studies could be embedded in computer science programs to help students explore diverse career options and understand the broader societal implications of their work. Similarly, simulations centered on green economy competencies could be used in business programs to prepare students for sustainable business practices and evolving market demands. Furthermore, universities could implement CA certificate programs by acknowledging the four core CA dimensions—confidence, control, curiosity, and coping. These may include a requirement for students to complete at least two industry co-designed projects under the joint supervision of academic staff and enterprise mentors.

Further supporting this approach, Wang et al. (2024b) demonstrated the efficacy of structured career interventions through a randomized controlled trial using the Satir Growth Model (SGM). Their study showed that a seven-session SGM-based career intervention significantly enhanced career exploration and adaptability among Chinese college freshmen, with career exploration serving as a partial mediator. Notably, these positive effects were sustained at a one-year follow-up, indicating the long-term utility of such programs (Wang et al., 2024b). These findings reinforce the value of incorporating psychological and growth-oriented frameworks into career development initiatives.

By adopting this integrated approach, disciplinary instructors can become key attachment figures who not only impart academic knowledge but also serve as secure bases for career exploration. Their dual role as educators and mentors enables them to provide emotional containment (e.g., reassurance during periods of uncertainty) and professional scaffolding (e.g., industry insights and reality testing), both of which are critical for shaping students’ internal working models of career competence and confidence. This strategy not only enhances CA but also strengthens future work self-clarity, thereby directly addressing the skill-mismatch-induced “slow employment” challenge.

#### 5.2.2 Implementation of phase-tailored career intervention frameworks

A significant limitation of current career training systems is their disproportionate emphasis on senior-year students, leaving freshmen and sophomores with insufficient support. A phase-tailored intervention framework aligned with CCT is proposed to address this developmental gap. In Years 1–2, the focus should be placed on self-clarity development through the use of digital twin simulations of emerging occupations, enabling students to explore diverse career paths and grasp evolving industry trends. In Year 3, the emphasis should shift to decision-making capacity by implementing industry-academia rotational modules, such as a three-week program that includes 1 week of enterprise immersion, to provide practical experience and support informed career decisions.

In Year 4 and beyond, building transition resilience through anti-fragility training programs designed to help students normalize career uncertainty using cognitive restructuring techniques should be prioritized. Additionally, “slow-employment” buffer programs offering government-subsidized professional micro-certifications during job search periods should be introduced to maintain skill relevance and enhance employability. These initiatives are intended to reinforce the internal working models of adaptability and resilience of students.

#### 5.2.3 Developing teacher training programs that are adapted to PGO

Current career-related instruction frequently applies standardized support strategies that fail to account for individual differences in the PGO of students. However, this study found that PGO negatively moderates the relationship between CRTS and CA, indicating that high-PGO students may avoid seeking help to conceal perceived weaknesses, whereas low-PGO students may under-utilize available support due to low perceived relevance. To reduce the fear of negative evaluation and encourage help-seeking among high-PGO learners, anonymized help-seeking mechanisms, such as digital Questions and Answers platforms with optional identity concealment, should be implemented. A relationship-first approach is recommended for low-PGO students, beginning with trust-building sessions before delivering career guidance. Additionally, the use of gamified skill badges can reinforce incremental achievements, thereby enhancing motivation and increasing the perceived value of support services.

The suppressive effects of PGO on the pathway linking teacher career support to CA can be mitigated by tailoring support strategies to students’ motivational profiles, while simultaneously leveraging students’ intrinsic motivations and reinforcing secure attachment dynamics.

#### 5.2.4 Establishment of a dual-channel system for teacher development

The current shortage of career educators with integrated theoretical and practical expertise presents a major barrier to effective career education. To address this, a dual-channel teacher development system should be implemented.

Discipline faculty channel: Annual 120-h industry immersion programs for subject teachers should be implemented alongside competency-based pedagogy certification. Faculty efforts should be recognized and incentivized by integrating career education outcomes into promotion and evaluation criteria.

Professional career coaching track: Career coaches should have a minimum of 3 years of industry experience and a career development instructor license, along with specialized training to equip them with the skills needed to deliver targeted adaptability interventions and support services.

This dual-channel approach ensures that academic instructors and professional career staff are adequately equipped to serve as attachment figures, providing students with emotional security and professional insight as they navigate the complexities of modern career development.

## 6 Limitations and directions for future research

### 6.1 Limitations

Although this study offers valuable insights into the mechanisms linking CRTS, CA, and FWSS among university students, several limitations must be acknowledged, highlighting promising directions for future research.

First, the study is subject to causal inference and methodological limitations. The use of a cross-sectional design and reliance on self-reported measures restricts the ability to draw causal conclusions and may introduce common method bias. Longitudinal or experimental research designs should be employed to strengthen the validity and robustness of future findings, along with the integration of multi-source data, such as teacher evaluations or objective behavioral indicators.

Second, concerns regarding generalizability and cultural context must be acknowledged. As the sample was drawn exclusively from Chinese universities, the findings’ applicability to other cultural or educational settings may be limited. Future research should replicate this model across diverse cultural and institutional contexts to determine whether the observed relationships among teacher support, CA, and FWSS are culturally universal or context-dependent. Third, the unexpected finding that PGO exerted a stronger direct effect on CA than teacher career support warrants further investigation. This result indicates that individual motivational orientations may play a more influential role in shaping adaptive capacities than external support. Future research should explore this dynamic more deeply, potentially by examining interactions between PGO and other motivational constructs, such as intrinsic motivation or self-efficacy, or by employing qualitative methods to gain insight into students’ subjective experiences and decision-making processes.

The third limitation concerns the measurement of socioeconomic context. Although university type was controlled as a composite proxy, the study lacked direct measurement of individual-level family SES (e.g., parental education, family income) and a more nuanced regional economic indicator. The variable regional GDP presents a conceptual challenge, as it is unclear whether the household registration region or the university region is more theoretically relevant to the constructs of career adaptability and future work self. While our proxy is justified, its imperfection means we cannot fully rule out residual socioeconomic confounding. This could mean that the estimated effects of CRTS, while significant, might be partially inflated by these unmeasured factors. Future research would benefit from more sophisticated multi-level modeling that incorporates economic data from both regions, or from collecting individual SES data using less intrusive methods (e.g., broad categorical scales) to better disentangle these effects.

Fourth, this study has limitations regarding its sampling strategy. Although stratified random sampling was employed to ensure the inclusion of students from diverse socioeconomic backgrounds and institutional types across China, the within-university sample size was relatively small (ranging from 8 to 95 participants per university). Furthermore, to ensure statistical power and capture institutional heterogeneity, the proportional representation of our sample across different tiers and genders was not strictly aligned with the actual national university student population proportions. This results in potential under-representation of certain subgroup students. The findings are therefore more suitable for understanding the underlying psychological relationships among variables than for providing precise descriptive estimates of the absolute levels of CA or FWSS within the national student population.

The proposed model can be expanded by incorporating additional mediators, moderators, and intervention strategies. Although CA served as the primary mediating variable in this study, future research could examine other psychological mechanisms, such as career identity or self-efficacy, in decision-making. Moreover, this study highlighted a critical measurement challenge regarding the assessment of PGO. The modest convergent validity and model fit of the PGO scale remind us that the non-significant moderating effect found in H7 may partly reflect methodological limitations rather than the absence of a true theoretical effect. Future studies should thus develop and employ more robust, contextually adapted measures of PGO—or complement quantitative tools with qualitative approaches—to more accurately capture its nuanced role. Investigating alternative moderators, such as proactive personality or cultural values, could offer a more nuanced understanding of individual differences in career development. Finally, developing and evaluating targeted interventions, including PGO-adaptive teacher training or CA enhancement workshops, may yield both theoretical insights and practical tools for fostering career readiness among students.

### 6.2 Directions for future research

In light of the identified limitations, this study proposes several directions for future research.

First, future studies should adopt longitudinal or experimental approaches and incorporate multi-source data collection methods to address the limitations of the cross-sectional design and reliance on self-reported data. For example, researchers could design studies that gather input from both students and teachers through surveys or interviews. This would help establish causal relationships among career support, CA, and future work self-clarity among teachers while also reducing potential biases associated with single-source data.

Second, because the current sample was drawn from Chinese universities, the generalizability of the findings to other cultural or educational contexts may be limited. Therefore, future research should replicate the proposed model in more diverse cultural settings to determine whether the relationships among teacher support, CA, and FWSS are culturally universal or context-specific. Cross-cultural comparative studies could offer valuable insights into how cultural values, educational systems, and societal expectations shape students’ career development processes.

Third, an unexpected finding of this study was that university students’ PGO exerted a stronger direct effect on CA than teacher career support, indicating that in shaping students’ adaptive capacities, individual motivational orientations may play a more pivotal role than external resources. Future research should explore the interaction between PGO and other motivational constructs, such as intrinsic motivation, self-efficacy, or achievement goal orientations, to better understand this phenomenon. Moreover, qualitative or mixed-methods approaches could be employed to capture students’ subjective experiences, perceptions of teacher support, and the psychological mechanisms through which PGO affects career development.

Fourth, future research should consider employing a probability-proportional-to-size (PPS) sampling design to enhance the representativeness and diversity of the sample. By using PPS sampling, researchers can ensure that each stratum (e.g., different universities, socioeconomic backgrounds) is adequately represented in proportion to its size in the population. This approach will allow for larger within-stratum samples, which can better capture the variability and characteristics of each subgroup. Such a design would not only improve the generalizability of the findings but also provide more precise estimates of CA and FWSS across different segments of the university student population. This methodological refinement is crucial for obtaining a more comprehensive understanding of the underlying psychological relationships and for developing targeted interventions to support university students’ career development.

Future research should also move beyond examining single sources of support and instead develop integrated models that investigate the concurrent influences of both teacher and parental support. This attachment theory framework underscores the importance of multiple attachment figures in a student’s development. However, the current study focused primarily on the academic sphere. Given emerging longitudinal evidence that career-specific parental support uniquely predicts students’ proactive career behaviors (Wang et al., 2024a), it is critical for future studies to incorporate familial dynamics. It is specifically recommended that researchers integrate both teacher and parental support within a unified model to examine their complementary yet distinct roles. This line of inquiry would clarify whether these influences operate additively, competitively, or synergistically in fostering the CA resources and FWSS of university students, thereby offering a more holistic understanding of the developmental ecosystem that shapes career development.

Fifth, while CA was identified as a key mediator in this study, future research should investigate additional mediating mechanisms, such as career identity, career decision-making self-efficacy, or proactive personality, that may further explain how teacher support influences career outcomes. Additionally, examining other potential moderators, such as cultural values, socioeconomic status, and educational background, could clarify the model’s boundary conditions and enhance its explanatory power. These expanded insights could inform the development and evaluation of targeted interventions, such as PGO-adaptive teacher training programs, CA-focused workshops, or motivation-aligned career counseling strategies, thereby offering practical tools to support students’ career development and address the increasingly prevalent issue of “slow employment.”

## 7 Conclusion

This study investigated the influence of career support on the career development of university students, emphasizing the mediating role of CA and the moderating role of PGO. Drawing on attachment theory, teachers were conceptualized as dual-role attachment figures whose professional identity and caregiving functions jointly provide a secure base for students’ career exploration. The findings demonstrated that teacher career support significantly enhances students’ CA, which subsequently reinforces their FWSS. Furthermore, PGO moderated these relationships: students with high PGO exhibited stronger effects of CA on FWSS but weaker effects of teacher support on CA. This study theoretically contributes by extending attachment theory to the domain of higher education career development and by offering empirical support for attachment-informed career interventions. The results highlight the need for universities to embed CA training into disciplinary curricula, implement phase-tailored intervention frameworks, design PGO-adaptive teacher training, and establish a dual-channel teacher development system to support students in navigating the evolving labor market.

## Data Availability

The raw data supporting the conclusions of this article will be made available by the authors, without undue reservation.
